# What Is Carcinoid Syndrome? A Critical Appraisal of Its Proposed Mediators

**DOI:** 10.1210/endrev/bnad035

**Published:** 2023-12-01

**Authors:** Merijn C F Mulders, Wouter W de Herder, Johannes Hofland

**Affiliations:** ENETS Center of Excellence, Section of Endocrinology, Department of Internal Medicine, Erasmus MC & Erasmus MC Cancer Institute, 3015 GD Rotterdam, The Netherlands; ENETS Center of Excellence, Section of Endocrinology, Department of Internal Medicine, Erasmus MC & Erasmus MC Cancer Institute, 3015 GD Rotterdam, The Netherlands; ENETS Center of Excellence, Section of Endocrinology, Department of Internal Medicine, Erasmus MC & Erasmus MC Cancer Institute, 3015 GD Rotterdam, The Netherlands

**Keywords:** carcinoid syndrome, serotonin, bradykinin, tachykinins, catecholamines, histamine

## Abstract

Carcinoid syndrome (CS) is a debilitating disease that affects approximately 20% of patients with neuroendocrine neoplasms (NEN). Due to the increasing incidence and improved overall survival of patients with NEN over recent decades, patients are increasingly suffering from chronic and refractory CS symptoms. At present, symptom control is hampered by an incomplete understanding of the pathophysiology of this syndrome. This systematic review is the first to critically appraise the available evidence for the various hormonal mediators considered to play a causative role in CS. Overall, evidence for the putative mediators of CS was scarce and often of poor quality. Based on the available literature, data are only sufficient to agree on the role of serotonin as a mediator of CS-associated diarrhea and fibrosis. A direct role for tachykinins and an indirect role of catecholamines in the pathogenesis of CS is suggested by several studies. Currently, there is insufficient evidence to link histamine, bradykinin, kallikrein, prostaglandins, or motilin to CS. To summarize, available literature only sufficiently appoints serotonin and suggests a role for tachykinins and catecholamines as mediators of CS, with insufficient evidence for other putative mediators. Descriptions of CS should be revised to focus on these proven hormonal associations to be more accurate, and further research is needed into other potential mediators.

## Graphical Abstract

**Graphical abstract:**
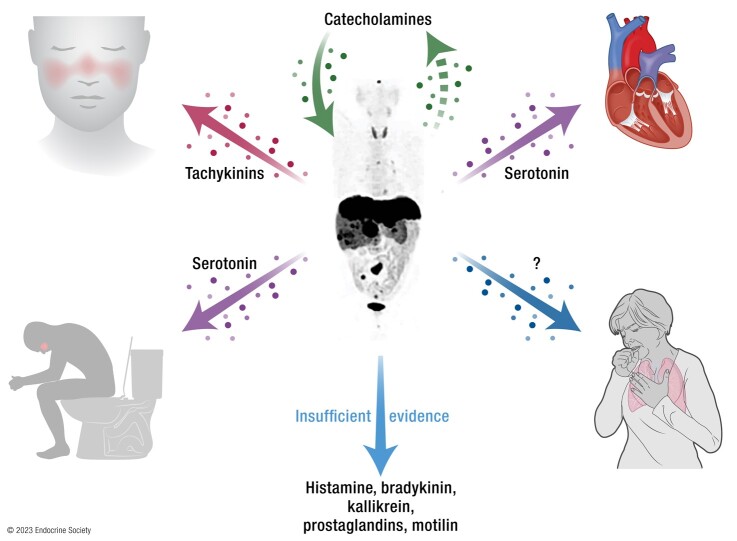

**Essential Points**

- Research on the various putative mediators of CS, the most prevalent hormonal syndrome in patients with neuroendocrine tumors, is surprisingly scarce and frequently of poor quality, leading to an incomplete understanding of its pathophysiology.
- While serotonin is likely the main causative mediator of diarrhea and fibrosis in the context of carcinoid syndrome, an inconsistent correlation with flushing and incomplete responses to serotonin pathway inhibitors suggest the involvement of other mediators in CS-associated flush.
- Available literature points toward a direct role for tachykinins and an indirect role of catecholamines in causing CS symptoms.
- Currently, there is insufficient evidence to link histamine, bradykinin, kallikrein, prostaglandins, motilin, and other putative mediators to CS, even though medications that can regulate these mediators are still either recommended or generally used to treat patients with CS.
- Descriptions of CS should be revised to focus on these proven hormonal associations to be more accurate, and further research is needed into other potential mediators.

## Background

Carcinoid syndrome (CS) is the most prevalent hormonal syndrome in patients with neuroendocrine neoplasms (NEN) (1, 2). CS was first characterized by Postma in 1927 and is currently defined by chronic diarrhea and/or flushing in the presence of systemic elevated levels of serotonin or its metabolite 5-hydroxyindolacetic acid (5-HIAA) (3, 4). At present, CS is estimated to affect 19% of all NEN patients, predominantly occurring in patients with well-differentiated grade 1 or 2 tumors with a small intestinal or bronchopulmonary origin (2). Flushing and diarrhea are the most distinctive features of CS and are present in 65% to 78% and 67% to 73% of CS patients, respectively (5, 6). Other, less prevalent symptoms of CS include bronchospasms, which are present in 8% to 10% of patients, and long-term fibrotic changes of the cardiac valves [carcinoid heart disease (CHD)] and mesentery (5-7). CHD occurs in 17% to 21% of CS patients and involves the right-sided cardiac valves in the vast majority of cases (5, 6, 8). This complication is a risk factor for right-sided heart failure, and early valve replacement is recommended for this patient category (9). Mesenteric fibrosis is estimated to be present in 70% of CS patients and leads to postprandial abdominal pain in the majority of patients, and prophylactic surgery should be considered to prevent obstructive complications (10, 11). Carcinoid crisis is viewed as an acute onset of hemodynamic instability or bronchospasms associated with CS, often occurring intraoperatively or during interventions (12, 13). It is estimated to occur in 19% to 35% of patients with a well-differentiated NET undergoing surgery and is generally viewed as the extreme end of the CS spectrum (12, 14). However, a recent prospective study did not observe a difference in the prevalence of carcinoid crisis between patients diagnosed with or without CS (12).

Patients with CS have a shorter overall survival and a worse quality of life compared to NEN patients without this syndrome (2, 15, 16). Due to the increasing incidence of NEN and improved overall survival of patients with these tumors over recent decades (17, 18), an increasing number of patients will experience CS symptoms for substantial periods of time. Despite the availability of first-generation somatostatin analogues (octreotide, lanreotide) and, more recently, telotristat ethyl, a substantial subset of patients with CS experiences treatment-refractory symptoms. Regretfully, little progress has been made in reducing the morbidity and mortality accompanying this syndrome of hormonal excess (19). Various putative mediators, including serotonin, catecholamines, brady- and tachykinins, kallikrein, histamine, motilin, and prostaglandins, have been suggested as potential causative factors for the symptoms and complications associated with CS (20-22). It is generally believed that most of these tumor-derived secretory products are inactivated by hepatocytes and, therefore, only cause symptoms when they either bypass or are secreted outside of the portal vein drainage system (7, 22). Pulmonary metabolism of these mediators also plays a role, as evidenced, for instance, by the right-sided predominance of CHD (8). However, the exact role of each potential mediator in the syndrome as a whole or within individual patients remains elusive. A better understanding of the pathophysiology of CS is needed to reduce the disease burden of this syndrome in treatment-refractory patients. We have chosen to use the systematic review approach to critically appraise the evidence for the various putative mediators of CS.

**Literature search**

EMBASE, MEDLINE, Web of Science, Cochrane CENTRAL, and Google scholar were systematically mined from inception to October 2022 to identify all research articles with evidence of hormonal mediators that possibly cause CS. Case reports and nonhuman studies were excluded. The search strategy is provided in the Supplementary Materials. This search yielded a total of 1477 hits. Thirty-two additional publications were added through the assessment of the reference lists of the included studies.

**Selection of studies**

**Figure 1.**
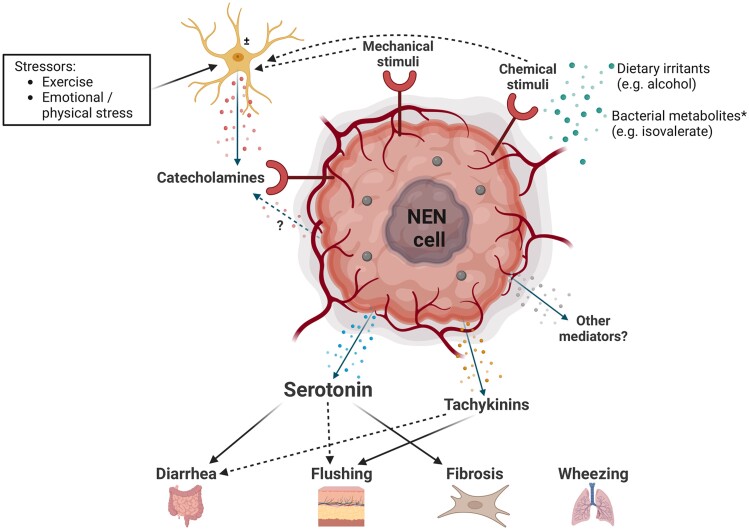
Proposed mechanism of the pathophysiology of the carcinoid syndrome. ±neuron/adrenal medullary cell. *Based on in vitro studies of an enterochromaffin cell organoid model. Solid line indicates strong association; dotted line indicates weak association (based on Tables 1 and 2). Created with BioRender.com. NEN, neuroendocrine neoplasm.

After removal of duplicates, 810 publications remained for eligibility check. Noneligible studies were excluded through title and abstract screening, resulting in 105 publications for full-text evaluation. Publications lacking quantitative outcome data specific for CS patients and those utilizing an nonspecific bioassay were excluded. This evaluation yielded a total of 74 articles that met the inclusion criteria for this systematic review. As most of the included studies were dated, there was a large variation in described assays, reference values, and even in the definition of CS. For the studies that were included in this review, the definition of CS and the assay reference values were adopted from the individual articles. Studies were evaluated according to the Preferred Reporting Items for Systematic Reviews and Meta-analyses statement (Fig. 1) (23).

Due to the heterogeneity of the included studies, a tailored, study-specific quality assessment was used instead of a standardized data extraction and quality control form. Study quality was independently assessed by 2 authors (M.M. and J.H.). Any inconsistencies between the 2 reviewers were checked by a third author (W.W.H.), who made the final decision on study quality.

### Serotonin

Serotonin [5-hydroxytryptamine (5-HT)] is synthesized by the enterochromaffin cells in the intestines (24). Thorson associated the symptoms of CS with elevated circulating levels of serotonin in 1954 (25). Over the decades to follow, various authors confirmed the presence of serotonin in NEN tissues, including 4 studies in CS patients (26-29). Many publications have demonstrated that levels of serotonin or its main metabolite 5-HIAA are regularly elevated in the circulation of CS patients. After exclusion of studies that defined CS by elevated serotonin concentrations (5, 30-33), 15 studies were identified that reported circulating or urinary serotonin or 5-HIAA levels specifically in NEN patients with clinical symptoms befitting CS (26-29, 34-44). When combined, routinely measured concentrations of serotonin and/or 5-HIAA in whole blood, serum, plasma, platelets, or (24-hour) urine were elevated in 98% (190/194) of these patients compared to healthy controls. (Table 1, row 1).

**Table 1.** Available evidence on tumoral or circulating presence of hormonal mediators in carcinoid syndrome patients

Mediator	Presence in tumor tissue	Presence in the circulation
Serotonin	4 (26-29)*^a^*	15 (26-29, 34-44)*^a^*
Catecholamines	1 (41)*^a^*	2 (41, 45)*^b^*
Bradykinin	0*^d^*	3 (45-47)*^c^*
Kallikrein	1 (48)*^c^*	1 (48)*^c^*
Tachykinins	4 (49-52)*^a^*	13 (37, 39, 42, 49-51, 53-59)*^a^*
Histamine	0*^d^*	3 (31, 60, 61)*^b^*
Prostaglandin	0*^d^*	2 (62, 63)*^b^*
Motilin	0*^d^*	1 (64)*^c^*

The table signifies the number of studies identified in this category, with references to studies that were identified.

^*a*^Present in >75% of patients.

^*b*^Present in 25% to 75% of patients.

^*c*^Present in <25% of patients.

^*d*^No studies available.

The strongest evidence for the role of serotonin in CS is derived from studies that analyze the effect of serotonin pathway inhibitors, like serotonin receptor antagonists, or inhibitors of tryptophan hydroxylase, the rate-limiting enzyme in serotonin biosynthesis. Of the 9 studies that have investigated the effect of serotonin receptor antagonists in CS patients (53, 65-72), only 2 were prospective clinical trials, and none were placebo controlled. One trial included 12 patients with a pretreatment stool frequency of 5 to 10 times daily and reported a reduction of stool frequency of >50% in 6/12 patients on high doses of the 5-HT_2_ and 5-HT_1_ receptor antagonist cyproheptadine. The frequency of flushes decreased by >50% in only 2/12 patients treated with this drug (65). In the other study, the 5-HT_3_ receptor antagonist ondansetron at 8 mg 3 times a day improved diarrhea in 6/6 CS patients, while 0/5 patients experienced improvement in flushing (70). The recently introduced tryptophan hydroxylase inhibitor telotristat ethyl has been tested in a series of phase I to III, multicenter clinical trials in patients with CS refractory to somatostatin analogues (73-76). In these studies, 43% (32/73) of patients who were treated with the approved dose of telotristat ethyl (250 mg 3 times a day) reported a reduction in daily number of bowel movements of ≥30% for ≥50% of the treatment period. In comparison, 12% (9/74) of patients treated with placebo also reported such a reduction in bowel movements. The effect of telotristat ethyl on flushing, a secondary endpoint in this patient selection, was limited, with only 1 out of 4 studies reporting a statistically significant reduction of flushing episodes (*P* = .041, mean decrease of flushing episodes of 27%) (75).

Four studies measured serotonin concentrations before, during, and after spontaneous flushes or flushes provoked by pentagastrin. One study included 10 CS patients with >4 flushes per day and measured fasting plasma 5-HIAA and serotonin concentrations before, during, and after spontaneous flushes in both the external jugular and forearm veins (45). During flushing, both serotonin and 5-HIAA concentrations were significantly higher at both sampling sites in all patients. Studies of serotonin concentrations during flushes provoked by pentagastrin have shown contradictory results. While 2 studies reported an increase in serotonin in only 1/28 patients (measured in plasma and provoked by 0.6 µg/kg in the first study and measured in whole blood and provoked by 0.06 µg/kg in the second study) (38, 39), a third study described a rise in whole blood serotonin levels in 18/19 patients as well as a response of flushing in those treated with the nonselective 5-HT_2_ receptor antagonist ketanserin (53). In this study, intravenous administration of 10 mg of ketanserin 10 minutes before the administration of 0.6 µg/kg of pentagastrin resulted in a decrease of flush severity in 14/15 CS patients and abolishment of flushing in 7/15 patients.

Three studies objectively quantified flushes and/or diarrhea and correlated them with the height of circulating serotonin or urinary 5-HIAA concentrations in CS patients (38, 54, 77). A study among 168 CS patients described a significant correlation between circulating serotonin and frequency of bowel movements, while flushing was not significantly associated with circulating serotonin concentrations (77). The other 2 studies included smaller cohorts and reported a moderate correlation between the severity of diarrhea and levels of whole blood serotonin (rho = 0.46, compared with stool weight) or urinary 5-HIAA (rho = 0.4, severity defined as none, occasional, or daily) (38, 54). In these studies, a similar correlation was found between urinary 5-HIAA levels and flushing (rho = 0.4, severity defined as none, occasional, or daily), while a poor correlation was observed between the peak of flush severity and the peak of whole blood serotonin concentrations (rho = 0.21).

Eight studies investigated the association between circulating or urinary 5-HIAA levels and the presence of CS-associated fibrotic complications. These concentrations were significantly higher in CS patients with CHD or mesenteric fibrosis compared to CS patients without these fibrotic complications (10, 44, 78-81). Moreover, patients with more severe CHD had higher 5-HIAA levels in serum, plasma, and 24-hour urine compared to those with lower severity (30, 80, 82). Interestingly, no differences between right- and left-sided intracardiac plasma and platelet serotonin concentrations were observed in 1 study of 12 patients with carcinoid heart disease (44). A single prospective study was unable to relate serotonin to the occurrence of carcinoid crisis (12). This study by Condron and colleagues included 16 patients with CS and measured peripheral whole blood serotonin concentrations before, during, and after intraoperative carcinoid crises. While preincision serotonin concentrations were higher in patients with crises compared to those without, no significant differences were found between preincision and midcrisis serotonin concentrations. Available literature on this mediator and its role in the various symptoms of CS is summarized in Table 2.

**Table 2.** Available evidence of the association between hormonal mediators and carcinoid syndrome symptoms

Mediator	Correlation with diarrhea	Correlation with flushing	Correlation with fibrosis	Correlation with wheezing	Correlation with carcinoid crisis
Serotonin	16 (38, 53, 54, 65-77)*^a^*	18 (38, 39, 45, 53, 54, 65, 67, 68, 70-77)*^b^*	8 (10, 30, 44, 78-82)*^a^*	0*^d^*	1 (12)*^c^*
Catecholamines	1 (83)*^a^*	4 (45, 83-85)*^a^*	1 (86)*^b^*	0*^d^*	0*^d^*
Bradykinin	0*^d^*	3 (45-47)*^b^*	0*^d^*	0*^d^*	1 (12)*^c^*
Kallikrein	0*^d^*	1 (48)*^c^*	0*^d^*	0*^d^*	1 (12)*^c^*
Tachykinins	2 (54, 59)*^a^*	6 (39, 49, 53-55, 57)*^b^*	0*^d^*	0*^d^*	0*^d^*
Histamine	0*^d^*	2 (26, 35)*^c^*	0*^d^*	0*^d^*	1 (12)*^c^*
Prostaglandin	3 (59, 62, 63)*^b^*	0*^d^*	0*^d^*	0*^d^*	0*^d^*
Motilin	0*^d^*	1 (64)*^c^*	0*^d^*	0*^d^*	0*^d^*

The table signifies the number of studies identified in this category, with the references to studies that were identified.

^*a*^Evident association.

^*b*^Inconsistent association.

^*c*^No association.

^*d*^No studies available.

Based on effect of pathway inhibitors, rise after provocation, if infusion produces similar symptoms, and the correlation with severity of the symptoms.

### Catecholamines

As the carcinoid flush can be provoked by emotional and other stimuli of the sympathetic nervous system, catecholamines have been linked to CS. This relationship is supported by evidence of β-adrenoreceptors on NEN cells as well as the presence of catecholamines and the catecholamine-synthesizing enzymes monoamine oxidase and catechol-O-methyltransferase in tumor tissue of CS patients (41, 45, 87). Two studies reported routinely measured catecholamine concentrations in CS patients, using validated assays. In 1 report, concentrations of the (nor)adrenaline metabolite vanillylmandelic acid, determined in plasma drawn in a resting state, were 2.5-fold higher in 10 CS patients compared to healthy controls, while adrenaline and noradrenaline levels did not differ between these 2 groups (45). Another study reported elevated 24-hour urinary catecholamine metabolite concentrations in 12/20 CS patients with flushing compared to healthy controls (41) (Table 1, row 2).

The strongest evidence for catecholamines as a mediator of CS can be derived from 2 studies that analyzed the effect of adrenergic receptor blockade (83, 84). Nonselective blockade of the α-adrenergic receptors by intravenous phentolamine (0.6-1 mg/sec or 5-15 mg 5 minutes before 0.6-20 µg of adrenaline or 1-20 µg of noradrenaline) lead to prevention of (nor)adrenaline-induced flushes in 13/14 CS patients while preventing flushes induced by alcohol, hepatic massage, pain, and positional change in 5/7 patients. Oral administration of phentolamine (50 mg/4 hours) and phenoxybenzamine (10 mg/d), another nonselective α-adrenoreceptor antagonist, resulted in a reduction of the number and intensity of flushes in 11/13 patients. Moreover, a switch from phentolamine to placebo in the 2 patients who reported a complete relief of flushes resulted in a recurrence of flushes within 24 hours. These authors reported a subjective relief of diarrhea in “several patients,” without providing more specific data. Again, in an unspecified number of patients, subtherapeutic doses of the nonselective β-adrenergic receptor blocker propranolol (20 mg/8 hours PO or a bolus of 0.25 mg IV) were unable to block the adrenaline-induced flush (84). Treatment with these nonselective α- or β-adrenergic receptor blockers was discontinued due to intolerable side effects in the majority of patients.

Other evidence includes studies in CS patients with either provoked or spontaneous flushes. Across 3 studies, intravenous administration of a bolus of adrenaline (0.6-20 µg) or noradrenaline (1-20 µg) induced flushing in 31/31 CS patients (83-85). These flushes occurred markedly slower than those provoked by serotonin or bradykinin administration (40-120 seconds vs 10-40 seconds). In addition, another study reported a significant increase in plasma noradrenaline and its metabolite in the external jugular vein during spontaneous flushing as compared to preflush and antecubital vein measurements in 10 CS patients (45). Research into the role of catecholamines in CHD is limited to 1 study, which did not find differences in urinary catecholamine excretion in 10 patients with CHD compared to 10 CS patients without this fibrotic complication (86). However, specific parameters of cardiac stress, like N-terminal atrial natriuretic peptide and heart rate variability, were significantly correlated with urinary excretion of the adrenaline metabolite metanephrine. Available evidence for the correlation of this mediator with the various symptoms of CS is reported in Table 2.

### Bradykinin and Kallikrein

The vasoactive neuropeptide bradykinin has been proposed as the mediator of the carcinoid flush in early reports, which included 3 to 9 CS patients and utilized a nonspecific bioassay (84, 88-90). Furthermore, kallikrein, a protease that forms bradykinin from its precursor, was reported to be increased in hepatic metastases of CS patients (88). However, these results could not be replicated in later studies. A study that used a specific kallikrein assay did not find evidence for the presence of intratumoral kallikrein in the primary tumors of 6 CS patients, and routinely measured plasma kallikrein concentrations in 20 CS patients did not statistically differ from those measured in 17 controls (48). Additionally, the 8 patients who experienced flushing after alcohol ingestion showed no change in mean plasma kallikrein activity at 2, 5, and 10 minutes after ingestion. The 2 studies that measured bradykinin (1 in whole blood, the other in plasma) during spontaneous flushing did not find an increase in any of the 17 CS patients with 1 or more daily episodes of flushing (45, 46). In contrast, a study that measured whole blood bradykinin concentrations during flushing provoked by pentagastrin (0.6 mcg/kg) reported an increase of bradykinin levels of >100% in 4/11 CS patients (47). In these patients, baseline bradykinin concentrations did not differ from healthy controls (45-47). In their prospective study, Condron and colleagues did not observe a rise in plasma bradykinin or kallikrein concentrations during carcinoid crises (12). Available evidence is summarized in Tables 1 and 2.

### Tachykinins

The discovery of the simultaneous presence of tachykinins and serotonin within the enterochromaffin cell prompted authors to explore their role in CS (91). The confirmation of this finding in later studies (54, 92) and the presence of tachykinins in tumor tissue as well as in tumor cell culture medium of CS patients further supports a role for these vasoactive neuropeptides in CS (49-52). Thirteen studies of CS patients measured concentrations of either tachykinins combined or those of individual tachykinins, like substance P, neurokinin A, or neuropeptide K (37, 39, 42, 49-51, 53-59). When compared to healthy controls, CS patients had elevated tachykinin concentrations in 77% (79/102) of peripheral venous plasma samples, in 86% (6/7) of hepatic venous blood samples, and in 85% (41/48) of 24-hour urine samples. While substance P was elevated in the peripheral venous plasma of 42% (30/71) of CS patients, neurokinin A and neuropeptide K were elevated in 67% (14/21) and 75% (12/16) of CS patients, respectively (Table 1, row 5).

Plasma tachykinin concentrations before, during, and after spontaneous and provoked flushes were measured in 6 studies. In 1 study, tachykinin concentrations increased by >100% in 9/13 CS patients during flushes provoked by pentagastrin (0.6 mcg/kg IV) (55). Another study reported a median rise of tachykinin levels of 143% in 20 CS patients with identical flush provocation (39). During flushing provoked by the same dose of pentagastrin, substance P and neurokinin A increased in 5/24 and 3/7 CS patients, respectively (53, 55, 57). In a study investigating food-induced flushes, substance P and neurokinin A rose by >100% in 2/10 and 3/10 CS patients, respectively (56). During spontaneous flushing, tachykinin concentrations rose by 28% and 293% in 2 patients (49, 55). Two studies correlated tachykinin concentrations to the severity of the carcinoid flush and reported variable results. One study described a positive correlation of substance P and neurokinin A levels with the timing of a food-induced flush (rho 0.46 and 0.62, respectively) and a negative correlation with the rise in facial temperature (rho −0.59 and −0.57, respectively) in 10 CS patients (56). Another study reported a poor correlation between tachykinin concentrations and the frequency of flushing in 44 CS patients (rho = 0.3) (54). An association of tachykinins with diarrhea is supported by 2 studies. One study measured small intestinal tachykinin concentrations via an orally inserted 2-balloon tube and reported them to be significantly higher in 10 CS patients compared to healthy, sex-matched controls (59). The other study reported a significant correlation between plasma tachykinin concentrations and the frequency of diarrhea in 44 CS patients (rho = 0.6) (54). Available evidence for the correlation of this mediator with the various symptoms of CS is summarized in Table 2.

### Histamine and 5-hydroxytryptophan

A potential link of histamine with CS was first described by Oates and colleagues in 1962, who described an “atypical” CS associated with metastasized gastric NEN (type and grade not defined) (93). These patients would present with elevated urinary concentrations of histamine and 5-hydroxytryptophan (5-HTP), the precursor of serotonin, combined with clinical features of red, patchy flushes, markedly different from the regular carcinoid flush. Surprisingly, aside from case reports, only 1 study could be identified that mentioned such patients. This study of 27 CS patients only measured histamine concentrations in the 4 patients who had elevated circulating 5-HTP levels. In these CS patients (1 pancreas, 1 gastric, and 2 of unknown primary origin), 24-hour urinary histamine concentrations were increased in 2 patients and symptoms that resembled those described by Oates and colleagues were only described for the patient with the gastric NEN (type and grade not defined) (31). The only other study that measured 5-HTP in CS patients did not find elevated 24-hour urinary 5-HTP concentrations in 6 NEN patients with symptoms of flushing and diarrhea (3 ileum tumors, 3 tumors of unknown primary origin), 3 of whom reported episodes of wheezing (27). In these studies, concentrations of 5-HTP and histamine were compared with reference values obtained in healthy controls.

Three other studies measured histamine concentrations in CS patients, reporting contradictory results. While urinary histamine concentrations were elevated in 1/6 CS patients in 2 studies (controls not defined) (32, 60), plasma histamine concentrations were elevated in 8/8 patients compared to “control patients” in the third study (threshold upper limit of normal defined as 3 pmol/L) (61). The only study that analyzed the effect of histamine pathway blockers in more than 1 patient did not find an effect of pharmacological doses of the histamine-1 receptor blockers mepyramine (50-100 mg/6-8 hours IV, IM, and PO) and promethazine (50 mg IM) on flushing in 3 investigated patients (26). Provocation of 3 CS patients with an intravenous injection of 25 mg histamine acid phosphate induced a flush accompanied by a fall in blood pressure, in contrast to a rise in blood pressure, which occurred in spontaneous and adrenaline-induced flushes (35). The prospective study by Condron and colleagues was unable to associate plasma histamine concentrations with the occurrence of carcinoid crisis (12). While some authors have reported elevated intratumoral histamine concentrations in NEN tissue, no studies were found in which intratumoral histamine concentrations were measured in the tumor tissue of more than 1 CS patient. Moreover, no studies could be identified that correlated histamine concentrations to the wheezing associated with CS. Available evidence is summarized in the sixth row of Tables 1 and 2.

### Other Postulated Mediators of CS

Even though a large number of hormonal substances are mentioned in the context of CS, evidence for other putative mediators is limited to a handful of studies. Two studies measured prostaglandin concentrations in CS patients. While prostaglandin F in serum or plasma was elevated in 19% (6/32) of patients compared to healthy controls, plasma prostaglandin E was elevated in 63% of CS cases (20/32) (62, 63). Moreover, a study that measured small intestinal prostaglandin E concentrations via an orally inserted 2-balloon tube found them to be elevated in 9 CS patients compared to 10 healthy, sex-matched controls (59). These studies reported contradictory results when correlating prostaglandin concentrations to diarrhea severity in CS patients. While intestinal prostaglandin E concentrations were correlated to the frequency of diarrhea (rho = 0.8) in 1 study (59), the 2 other studies reported a poor correlation between circulating prostaglandin E and F concentrations and the severity of diarrhea (no definition of severity or correlation coefficient was provided) (62, 63).

Although motilin has been linked to diarrhea in NEN patients (94), only 1 study determined motilin concentrations specifically in CS patients (64). This study of 6 CS patients with daily spontaneous flushing showed that plasma motilin was not elevated before and during flushes provoked by noradrenaline, pentagastrin, and alcohol. A correlation with diarrhea was not analyzed in this study.

Measurements of calcitonin gene-related peptide, human chorionic gonadotropin (HCG)-α and -β, GH, and prolactin in serum or plasma were sporadically elevated in CS patients compared to healthy controls (95-97). No significant association between calcitonin gene-related peptide and flushing was found. During provoked flushes, no clear rise was detected in plasma or serum concentrations of vasoactive intestinal peptide, pancreatic polypeptide, GH, neurotensin, HCG-α, or HCG–β in CS patients (39, 64).

Available evidence is summarized in Tables 1 and 2.

### Combining Evidence for the Pathophysiology of CS

Despite the fact that CS was discovered over 70 years ago, there has yet to be a consensus on which mediators cause its symptoms. This is the first systematic review that critically appraises the available evidence for these mediators, and it shows that evidence for most of the proposed mediators of CS is extremely scarce and frequently of poor quality. Reviews and textbooks on the topic of CS have described up to 40 putative mediators associated with this disease (22, 98-100). However, in the past 2 decades, only a handful of studies were undertaken that have measured these mediators with contemporary sensitive and reliable assays after sampling of blood or urine under standardized conditions. Based on this review, evidence is only sufficient to agree on the role of serotonin as a direct mediator of CS. In addition, available data strongly suggest an indirect role for catecholamines in stimulating NEN cells to release the mediators of CS. Tachykinins likely also mediate CS symptoms, as they are frequently present in the circulation of CS patients and rise during provoked flushes in these patients. As summarized in Tables 1 and 2, hardly any evidence was found that implies a role for bradykinin, kallikrein, histamine, prostaglandins, motilin, or other putative mediators in the pathophysiology of CS.

Our results are in line with the recently published definition of CS and confirm that serotonin is its most well-established mediator (4). First, we found that serotonin was present in at least 98% of NEN patients with clinical symptoms befitting CS. Second, multiple prospective studies have shown that serotonin pathway inhibitors are able to significantly reduce diarrhea severity in CS patients. Additionally, we found a fairly good correlation between diarrhea severity and concentrations of circulating serotonin or 5-HIAA in CS patients. This association is broadly supported by preclinical studies that have investigated the effects of serotonin in the gastrointestinal tract (101). Serotonin functions through a complex network of 7 families of 5-HT receptors. In diarrhea, the 5-HT_3_ receptor appears to play an important role, as blockade of this receptor decreases diarrhea in patients with CS and other diarrheagenic syndromes (66, 67, 70, 101). Lastly, there is a strong body of evidence that supports a stimulatory role of serotonin in the fibrotic complications associated with CS. In addition to our findings and reports of endocardial fibrosis as a complication of serotonin agonist use (102-104), long-term administration of serotonin in rats is able to induce morphological changes resembling human CHD (105). This association has been confirmed in vitro, as studies have shown that fibroblasts express serotonin receptors and that serotonin receptor antagonists are able to reduce the secretion of profibrotic growth factors (106). Even though the effect of serotonin receptor blockade on fibrosis has not been studied in CS patients, the 5-HT_1a/b_ and 5-HT_2A/b_ receptors might be relevant treatment targets, as serotonin's effects on fibroblasts seems to be mediated via these receptors (107).

The fact that only 43% of CS patients reported a significant reduction of bowel movements after initiation of telotristat ethyl treatment, with no consistent effect on flushing, suggests the involvement of other mediators. Particularly flushing in CS patients has been incompletely understood thus far. Serotonin, tachykinins, and catecholamines have all been observed in the tumor tissue of CS patients and have been shown to exert vasoactive effects, pointing toward a potential role of these mediators in CS-associated flushing. A recent study investigated the physiology of intestinal neuroendocrine cells using an enterochromaffin cell organoid model and found that dopamine, epinephrine, and norepinephrine, but not histamine, bradykinin, and substance P, activated enterochromaffin cells to release serotonin (92). These authors showed that α2-adrenoreceptor antagonists ameliorated responses to all catecholamines, indicating the presence of adrenoreceptors on enterochromaffin cells. This hypothesis has been confirmed by in vitro and in vivo work of others and is supported by our analysis, as nonselective α-adrenoreceptor antagonists prevented adrenaline- and pressure-induced flushes in CS patients (83, 84, 87, 108). However, catecholamines induce vasoconstriction when bound to adrenoreceptors on the vascular wall (109), pointing toward an indirect role of catecholamines in the carcinoid flush through the induction of secretion of other hormonal mediators by NEN cells, rather than a direct stimulatory role of catecholamines on blood vessels. It is currently unknown whether the elevated catecholamine levels found in CS patients result from stimulation of the autonomous nervous system or from tumoral secretion. Both tachykinins and serotonin can induce vasodilatation through respectively binding to the NK1 and 5-HT_2B_ and 5-HT_7_ receptors, which are present on endothelial and smooth muscle cells of blood vessels (110-113). While studies measuring serotonin levels during spontaneous and provoked flushes in CS patients report contradictory results, tachykinin levels seem to rise more consistently during flushing in CS patients. Moreover, tachykinins have even been linked to the diarrhea present patients with CS (54, 59). Therefore, tachykinins could potentially contribute to the pathogenesis of the carcinoid flush and may even play a role in diarrhea in CS patients. However, a role for serotonin in the carcinoid flush cannot be excluded, as some studies do report a consistent rise in serotonin concentrations during flushing in CS patients (45, 53). Whether this rise in serotonin actually causes the flush or is merely simultaneously coexcreted by the tumor without a direct causative role remains to be elucidated. (Figure 1).

Surprisingly, although frequently named as causative mediators of CS, there was insufficient evidence to link mediators such as histamine, kallikrein, bradykinin, and prostaglandins to CS. There are several possible explanations for the inconsistent results regarding these mediators. Bradykinin and kallikrein were believed to be involved based on early studies that utilized a nonspecific bioassay. However, in the years to follow, these results could not be reproduced using more specific assays. Histamine is mainly believed to be involved in very specific subgroups of CS patients, such as those with episodes of wheezing or flushing in the context of an “atypical” CS associated with metastasized gastric NEN. As wheezing is only reported in 8% to 10% of CS patients (5, 6), and patients with CS rarely have a NEN of gastric origin (2), measurements of histamine concentrations in these patients are limited to case reports. Also, no evidence could be found for the involvement of other mediators of bronchospasms in CS patients. While prostaglandin E was quite consistently elevated in the 2 studies that measured this mediator in CS patients, the correlation between the prostaglandin E levels and the severity of diarrhea differed between these studies. This may be explained by the heterogeneity of the included patients. While one of the studies included a well-defined cohort of CS patients with a histologically proven ileal NEN (59), the other defined them as patients with “carcinoid tumors” (63). This is important because NEN of different origins may secrete different subsets and combinations of mediators. Recent research even suggests the existence of distinct enterochromaffin cell subtypes along the gastrointestinal tract, which contain different hormonal mediators and can be triggered by different stimuli (92, 114). This suggests that the composition of NEN cells causing CS can differ from patient to patient, explaining the inconsistent results of the included studies and supporting the lack of a typical hormone profile of CS patients in the single prospective study that utilized contemporary sensitive assays (12). Therefore, future research should focus on the possibility of multiple hormonal profiles within CS. Determining a comprehensive hormonal profile in well-defined subgroups of CS patients is essential to further elucidate its pathophysiology.

In conclusion, this systematic review shows that research on the various putative mediators of CS is surprisingly scarce and frequently of poor quality. While serotonin is likely the main causative mediator of diarrhea and fibrosis in the context of CS, an inconsistent correlation with flushing and incomplete responses to serotonin pathway inhibitors suggest the involvement of other mediators in the CS-associated flush. Available literature points toward a direct role for tachykinins and an indirect role of catecholamines. Evidence for other putative mediators was insufficient to reliably link them to CS. Future research should focus on the mechanisms that stimulate hormonal release in CS patients, as well as the possibility of multiple hormonal profiles within CS. Determining a comprehensive hormonal profile in various well-defined subgroups of CS patients is essential to further elucidate its pathophysiology, with the ultimate aim to more effectively treat CS in clinical care. In the meantime, descriptions of the hormonal mediators of CS should be limited to serotonin, tachykinins, and catecholamines to be more accurate.
